# Smartphone Step Data for Public Health: Insights From Surveillance in Japan and Future Perspectives

**DOI:** 10.2188/jea.JE20260059

**Published:** 2026-06-05

**Authors:** Takashi Nakagata, Yoshio Nakata

**Affiliations:** 1 Center for Physical Activity Research, National Institutes of Biomedical Innovation, Health and Nutrition, Osaka, Japan; 2 Microbial Research Center for Health and Medicine, National Institutes of Biomedical Innovation, Health and Nutrition, Osaka, Japan; 3 Institute of Health and Sport Sciences, University of Tsukuba, Ibaraki, Japan

Daily step count is an intuitive and widely understood indicator of physical activity.^1^ As wearable devices and smartphones have proliferated, step-based evidence has expanded, including dose–response meta-analyses linking daily steps to multiple health outcomes.^2^ In Japan, population monitoring of step counts has a long history: pedometer-based steps have been assessed in the National Health and Nutrition Survey since 1989.^3^^,^^4^ The Physical Activity Guide for Health Promotion 2023 provides step-count figures (8,000 steps/day for adults and 6,000 for older adults) as individual-level reference.^5^ To effectively promote physical activity, it is essential to identify specific periods throughout the day—from waking to bedtime—during which additional activity can be incorporated, and to establish concrete behavioral targets accordingly. In this context, Matsumoto et al report a timely nationwide longitudinal analysis of smartphone-measured steps in Japan that benchmarks changes during and after the April–May 2020 emergency declaration against a 2019 baseline.^6^ By resolving hour-by-hour patterns, the study moves the field beyond daily aggregates and clarifies when deficits were most pronounced—weekday commuting periods and late evening—helping to inform time-targeted public health strategies.

The direction and magnitude of step decline have been reported consistently in Japan (eg, municipal-level changes in Yokohama,^7^ occupational shifts,^8^ and regional/demographic heterogeneity^9^). Matsumoto et al extended this to a national diurnal lens that clarified which periods of the day drive aggregate change. The “valleys” appear consistent with exogenous structural constraints (reduced on-site work, curtailed evening venues), not autonomous behavior change alone. Although a causal inference lies outside the descriptive scope, the internal consistency across strata reinforces these structural explanations.

Matsumoto et al provide an example of descriptive epidemiology,^10^ combining the classic person–place–time framework with an unusually high temporal resolution. By resolving diurnal patterns across demographic groups (person) and urban contexts (place) over specific pandemic phases (time), it yields actionable insights into when and where to intervene. From a methodological perspective, diurnal metrics merit attention. Changes in the timing, amplitude, and regularity of daily step profiles offer sensitive indicators of disruption and recovery, which are obscured when relying on daily totals.

The larger declines among younger adults highlight differential constraints and preferences. Time- and place-targeted measures—such as commuter-hour prompts, improvements in evening walkability and lighting, and facilitation of venue access—may be particularly useful because they align with the times of day when deficits are greatest. This perspective aligns with Japan’s Active Guide,^11^ which encourages people to add 10 min of activity per day and reduce prolonged sitting. The Guide’s timeline-style framing, illustrating options during morning, midday, and evening and across three domains (lifestyle activity, exercise, and breaking up sitting), offers context-specific examples (eg, walking a child to school, active commuting, short movement breaks at work, or light exercises while watching TV). Such approaches should be paired with subgroup-sensitive messaging considering age, urbanicity, and socioeconomic and occupational characteristics.

From a surveillance perspective, such data streams complement established systems by offering near-real-time, high-frequency indicators. Device-derived measures provide scale, speed, and temporal granularity, which are valuable for routine policy evaluation and rapid situational awareness during crises. A recent country-wide natural experiment using smartphone step data (∼2.1 million users) reported that relocating from less- to more-walkable cities was associated with higher daily step counts (by ∼1,100 steps/day on average), sustained over months and consistent across strata of sex, age, and body mass index.^12^ The All of Us Research Program, using Fitbit wearable data, documented pronounced “activity inequality” by sex, age, body size, geography, and built environment, strongly correlated with obesity prevalence.^13^ Longitudinal analyses from All of Us showed that step-count volumes derived from participants’ own Fitbit devices are associated with incident chronic diseases across the phenome, with largely inverse associations for several outcomes and nonlinear patterns for diabetes and hypertension that show little additional association above ∼8,000–9,000 steps/day.^14^ Additional work from the same program suggests that novel wearable-derived metrics—such as daily heart rate per step—may show stronger associations with cardiovascular fitness and cardiovascular disease outcomes than steps alone.^15^ These results highlight the public health relevance and clinical potential of device-derived indicators at the national level.

As Matsumoto et al noted in their limitations, representativeness and the effect of periods when smartphones are carried versus not carried warrant attention; underestimation or overestimation due to non-carriage is well-documented.^16^ The authors appropriately discussed potential confounding by meteorological factors and limited generalizability from an app-based, self-selected user population. Cross-device calibration and algorithmic heterogeneity compromise comparability across platforms and times. Transparent reporting of device and operating system distributions, plausibility filters, calibration and sensitivity analyses, and cross-validation with traditional surveillance should become standard practices.

Matsumoto et al add an important piece to Japan’s coronavirus disease 2019 physical activity evidence base: not only that steps fell, but also when they fell and how incompletely they returned. Their diurnal signature supports time-targeted, place-aware countermeasures—such as commuter-hour messaging and improvements to evening environments—prioritized for subgroups most affected. The study also strengthens the case for incorporating device-based monitoring into routine surveillance. At the national level, integrating diurnal, device-derived indicators into Japan’s existing surveillance architecture—alongside questionnaire-based surveys and periodic accelerometer subsamples—could help provide a more responsive and resilient monitoring system. Establishing regional, hour-by-hour dashboards may be a useful future policy priority to link routine evaluation with rapid situational awareness. Combined with established governmental surveys and research-grade accelerometry, such systems could enhance Japan’s preparedness for future public health shocks.
